# How do medical students' expectations shape their experiences of well‐being programmes?

**DOI:** 10.1111/medu.15543

**Published:** 2024-09-24

**Authors:** Emmanuel Tan, Erik Driessen, Janneke Frambach, Jennifer Cleland, Grainne P. Kearney

**Affiliations:** ^1^ Lee Kong Chian School of Medicine Nanyang Technological University Singapore; ^2^ Department of Educational Development and Research, School of Health Professions Education (SHE) Maastricht University Maastricht The Netherlands; ^3^ Centre for Medical Education Queen's University Belfast Belfast UK

## Abstract

**Introduction:**

Medical schools have a duty of care to support their students' health and well‐being. Student support studies have tended to focus on outcomes in respect of effectiveness and satisfaction. In contrast, little is known about how student expectations of support may shape their experiences and engagement with support mechanisms, as well as the relationships students have with those offering support (including the institution itself). To address this gap in knowledge, we explore how medical students' expectations of student support systems shape, and are shaped by, institutional rules and processes.

**Methods:**

We employed a qualitative case study approach using an institutional ethnography informed methodology. Our context was a medical school that provides a well‐advertised, formal institutional support system where students are assigned a personal tutor. Data collection included interviews with medical students (*n* = 13) plus document analysis (public facing artefacts and internal policies/guidelines related to the support system). We applied the lens of psychological contract theory to guide abductive analysis of interview and text data.

**Results:**

Students expected a strong support system to be provided by the medical school and the personal tutors. However, their experiences did not always align with their expectations. Some felt excluded by the system while others regarded the relationship with their personal tutor as more transactional than relational. Where their expectations were unmet, students responded by reducing their engagement with the formal support system and creating their own peer‐support network that supplemented existing formal support.

**Discussion:**

Student expectations matter in shaping their experiences of support systems. Where expectations are unmet, students may disengage and/or find alternatives. This may be easier for some students compared with others. More understanding of the relationship between expectations and engagement with support can inform the development of institutional support structures that meet the needs of all students across time.

## Abstract 2

By contrasting institution‐led well‐being programmes with those started by students themselves, Tan et al. explore how expectations shape experience and how medical schools can do better

## 1 INTRODUCTION

Medical schools have a duty of care to support their students' health and well‐being. This support may be offered in various ways, through mechanisms such as personal tutors assigned to each student and/or educational interventions such as stress management and resilience training.^1^, ^2^, ^3^ The existence and format of such support mechanism(s) are usually conveyed to prospective and current students by public‐facing documents such as the medical school's webpages and handbooks for students. Via these communications, students form certain expectations (what an individual thinks they will receive from another party) on how the school will support their well‐being.^4^

Given expectations shape experiences and engagement with different aspects of education, it is likely that these also shape students' experience of, and engagement with, well‐being programmes and activities.^5^ For example, a mismatch between students' expectations and the (perceived) reality of support received may have detrimental effect on the effectiveness of support.^6^ Yet, to date, studies on well‐being interventions offered by medical schools have tended to focus on outcome measures such as programme efficacy or satisfaction.^7^, ^8^ There is a gap in knowledge in terms of understanding how expectations can affect how students interpret and make sense of the support they receive from their medical school and how this sense making shapes relationships with support staff, such as personal tutors, and the institution.

Psychological contract theory offers a useful framework to understand students' expectations and beliefs about formal support systems. The concept of the psychological contract was first introduced by Chris Argyris, who described it as a set of implicit and unwritten rules between employers and employees that influence productivity and grievance levels at work. Denise Rousseau further advanced this concept by emphasising the role of an individual's personal beliefs in shaping the reciprocal nature of the relationship between organisations and employees. Psychological contract is deeply rooted in social exchange theory,^9^ which posits that an individual can hold beliefs about their reciprocal relationship and implicit agreement with another party, based on promises and expectations.^10^ These promises can be both explicit, such as (in this case) commitments to student support published in webpages and handbooks. Promises can also be implicit through, for example, organisation culture and institutional rules and practices.

These tacit agreements are dynamic, with individuals making their own assumptions when their expectations do not align with their experiences. The assumptions and how they might change over time can, in turn, influence individuals' attitudes and how they behave in relation to other people.^11^ As such, the employee's (or student's) cost–benefit analysis of the relationship may influence their behaviours and commitment to the organisation (in this case, medical school).

The psychological contract is thus a useful framework for understanding the nuanced and dynamic nature of the employer–employee (student–medical school) relationships. It highlights the importance of managing expectations and perceptions to foster a productive and harmonious work or educational environment. Similarly, it explains how incongruence in the psychological contract can lead to disengagement, loss of trust in the relationship and unsatisfactory outcomes^12^, ^13^ such as exit (leaving the relationship), voice (actively showing disappointment), loyalty (passively waiting for situation to improve) and/or neglect (reducing commitment and allowing the situation to deteriorate).

Studies from wider higher education have explored the role of the psychological contract on the work values of students,^14^ student happiness,^15^ diversity issues in the classroom,^16^ student motivation and engagement,^17^ academic supervision^18^ and the psychological contract between students and tutors.^19^ Of particular interest is a recent paper^20^ examining the use of psychological contract in psychology student–personal tutor relationships. The study found that ‘breaches’ of psychological contract between students and their personal tutors (e.g. tutor unavailability) led to negative emotional and behavioural responses, such as students feeling isolated and disregarded, and turning to other staff for help instead of their assigned tutors. Student interactions with their personal tutors influenced (both negatively and positively) the relationships between student and tutor, and student and institution.

In this paper, we report on a case study that explores medical students' expectations and experiences of a formal well‐being programme. We use psychological contract as a theoretical lens to unpack the relationship between the students and their personal tutors. To this end, we ask: How do medical students' expectations of a student support system shape, and are shaped by, institutional rules and processes?

## 2 METHODS

### 2.1 Study design

We used institutional ethnography (IE) in an informed way^21^ as our methodological approach. IE focuses on social organisation, institutional discourses and power relations, key aspects in unpacking how institutional expectations (in this case, expectations of a support programme) are communicated and enacted within the institutional context. Using IE in an informed way (with specific focus on work, texts and discourse, see below) allowed us to be pragmatic in our data collection and analysis. We used semi‐structured interviews and document analysis, and the interaction between these, to understand the work (the activities that medical students engaged with in relation to student support within the institution), texts (the documents connected to student support within the institution) and discourse (dominant language practices within the institution) in the medical school and its student support programme.

### 2.2 Case setting

A case study approach is designed to provide an in‐depth exploration of the complexities and intricacies of a particular phenomenon within its real‐life context.^22^, ^23^ The specific context of our case study was one of Singapore's three medical schools, Lee Kong Chian School of Medicine (LKCMedicine), Nanyang Technological University Singapore. At the time of data collection, the LKCMedicine 5‐year programme was a partnership with Imperial College London and thus modelled on the traditional UK system of mostly undergraduate entry to medicine. Our students are mostly aged between 18 and 25 years of age (see previous studies^1^, ^24^ for more background information). LKCMedicine has a ‘house system’, where students are stratified by gender and high school, then randomly assigned into one of the five ‘houses’ for student support.^24^ The primary purpose of the house system is to support student well‐being. The ‘house’ is symbolic rather than physical, a way of organising student well‐being activities. Each student is also assigned a house tutor, whose main responsibility is to provide student support/pastoral care and who is not involved in assessment or academic progression of students. The house tutors consist mainly of general practitioners in private practice (about half of the team), clinicians from Singapore's public hospitals and faculty members from the institution. Students typically meet their tutors four times a year for one‐to‐one meetings.

Students are informed about the house system via multiple ways. For example, for prospective students, the house system is featured prominently during open house events, on the school webpages and student presentations. Following their enrolment in the school, the house system framework is explicitly explained to students, and well‐being activities, such as interhouse games and social events, are formally scheduled by the medical school. The house system type of student support is now implemented in all Singapore's medical schools and is seen in other medical schools worldwide.^25^, ^26^, ^27^

### 2.3 Participants

We invited medical students from all 5 years to participate in the study via email. Our aim was to recruit 10–15 interviewees, anticipated to be sufficient to address our research question^28^ given the relatively homogeneous student population, the aim of the study, our use of established theory and approach to analysis.^29^, ^30^ We purposively sampled from different years, genders, and houses to gain a rich picture of how the psychological contract is formed in relation to student support. Our aim in doing so was to gain an in‐depth exploration of the students' experiences and relationship with their house tutor and the house system programme.

### 2.4 Data collection and analysis

#### 2.4.1 Interviews

Individual interviews were conducted by E. T. (see later for positionality) using a semi‐structured interview guide designed by drawing on our knowledge of the student support and psychological contract literature (see Appendix S1). Thirteen participants participated, with eight identifying as male and five as female, reflecting the gender split generally representative of the wider student population. Participants were almost evenly distributed across Years 1–4 students (Year 5 students had graduated at the time of data collection) and were mainly drawn from three of the five houses. The average interview duration was 40 minutes. We first asked students to talk about their general experiences of the house system. We then asked participants specific questions to elicit their views on the perceived promises, expectations and obligations of the psychological contract with questions such as: What do you think is the role of a house tutor? How strongly do you identify as a member of your house? The interview questions were intentionally drafted to elicit participants' exploration of institutional promises, expectations and obligations, rather than directly asking them about the documents in which these were presented. In other words, we focused on what actions the documents brought about (their work) as opposed to considering the documents in isolation.

#### 2.4.2 Documents

In line with IE methodology, we collected public facing artefacts and documents, such as the school webpage referring to support and the documents which present the house system, including its role in key school events and ceremonies. We also gathered internal texts such as the house tutor guide, house system handbook for students, and student agreement (see Appendix S2) in order to further our understanding of how the relationship between the students and the school is coordinated and organised.^31^ These documents included information on the support structure within the institution (house system guide) and the expectations and responsibilities of students (student agreement). The concern form for medical students, mitigating circumstances form and other student‐related documents outlined key student life and academic processes. Finally, the minutes of the White Coat Ceremony, Student Well‐being Committee meetings and Student–Staff Liaison Committee meetings offer records of significant events and discussions around student life and well‐being.

The interview data were transcribed verbatim, anonymised and analysed using an inductive approach. Two members of the research team (E. T. and G. K.) first independently coded two transcripts, mapping out how students engaged with the house system, their house tutors and peers (the work of the students). This initial coding was guided with a focus on institutional promises and its impact on students' expectations of the support system. Using the notion of a psychological contract as a sensitising concept^32^ provided us with a general sense of reference and guidance in approaching the empirical data, helping us to abductively code key texts (e.g. institutional policies such as house system handbook for students)^33^ for institutional processes and rules that influenced students' interaction with their house tutors and participation in the house system activities. Key extracts were identified and compiled. Following further team meetings where the initial coding was discussed and debated, E. T. used the identified codes as a framework to analyse the remaining transcripts and documents. The analysis continued in an iterative and abductive fashion, via regular meetings and email discussions until the team reached a consensus on the final interpretation of the dataset.

#### 2.4.3 Reflexivity

Qualitative research is dependent on the relationship between the researcher and the research process (e.g. the literature^34^, ^35^). We considered our positions and relationships with the data continually and critically in view of our disciplinary backgrounds, levels of knowledge and perspectives on student support and qualitative research. E. T. and J. C. are insiders (‘emic’), contributing contextual knowledge from their leadership roles within the medical school (J. C. is Vice‐Dean of Education and E. T. is the Assistant Dean of Student Well‐being), as well as qualitative research expertise. E.D., J. F. and G. K. are outsiders ‘etic’, providing valuable critical views and qualitative research expertise. E. T. maintained a reflection journal. The research team met regularly, incorporating reflection in the meeting agendas to critically reflect on their positionalities and experiences, and their potential influences on the data, collection, analysis and findings. For instance, the team explicitly discussed E. T. and J. C.'s views of the house system, which they did not view as something negative or positive, but as something, they genuinely wanted to understand better.

The power differential between the authors and participants was a critical consideration in the interview data collection. E. T. inherently possessed authority and influence within the medical school. Yet the nature of his role is student support, and routine student feedback indicates they find him very approachable and someone who listens without judging. In addition, we were deliberate in our approach to ensure that participants felt comfortable during the interviews. E. T.'s reflection journal and our regular team meetings with reflexivity discussions were also important in considering and mitigating any potential biases.

## 3 RESULTS

We found that medical students formed expectations of the student support system very early on. These initial impressions were reinforced through their time in medical school. We identified tensions in the relationship between the students and the house system when the students' expectations were not fully met. Students engaged in sensemaking between what they understood and expected of the house system, and what they experienced. Additionally, students demonstrated agency. They responded to these tensions by creating workarounds, such as creating their own peer‐support network that supplemented existing formal support.

We structure our results along the main themes of what we found in the psychological contract between the students and the house system: the promises, sensemaking, reciprocity and, finally, empowerment and legitimacy.

### 3.1 Of promises and expectations

Participants felt that the house (well‐being/personal tutor) system was helpful in many ways. They highlighted that the house system allowed them to make friends and find peer support as well as receive support from a staff member. While stating ‘there's definitely a limit to what a House Tutor can do’ (P07), they sought guidance from their house tutors on a variety of matters, from routine administrative queries to more complex, personal support issues. As one participant put it, the house tutor was ‘someone you could go to if you face issues within school that are like, something that you don't know how to handle’. (P05).

**FIGURE 1:**
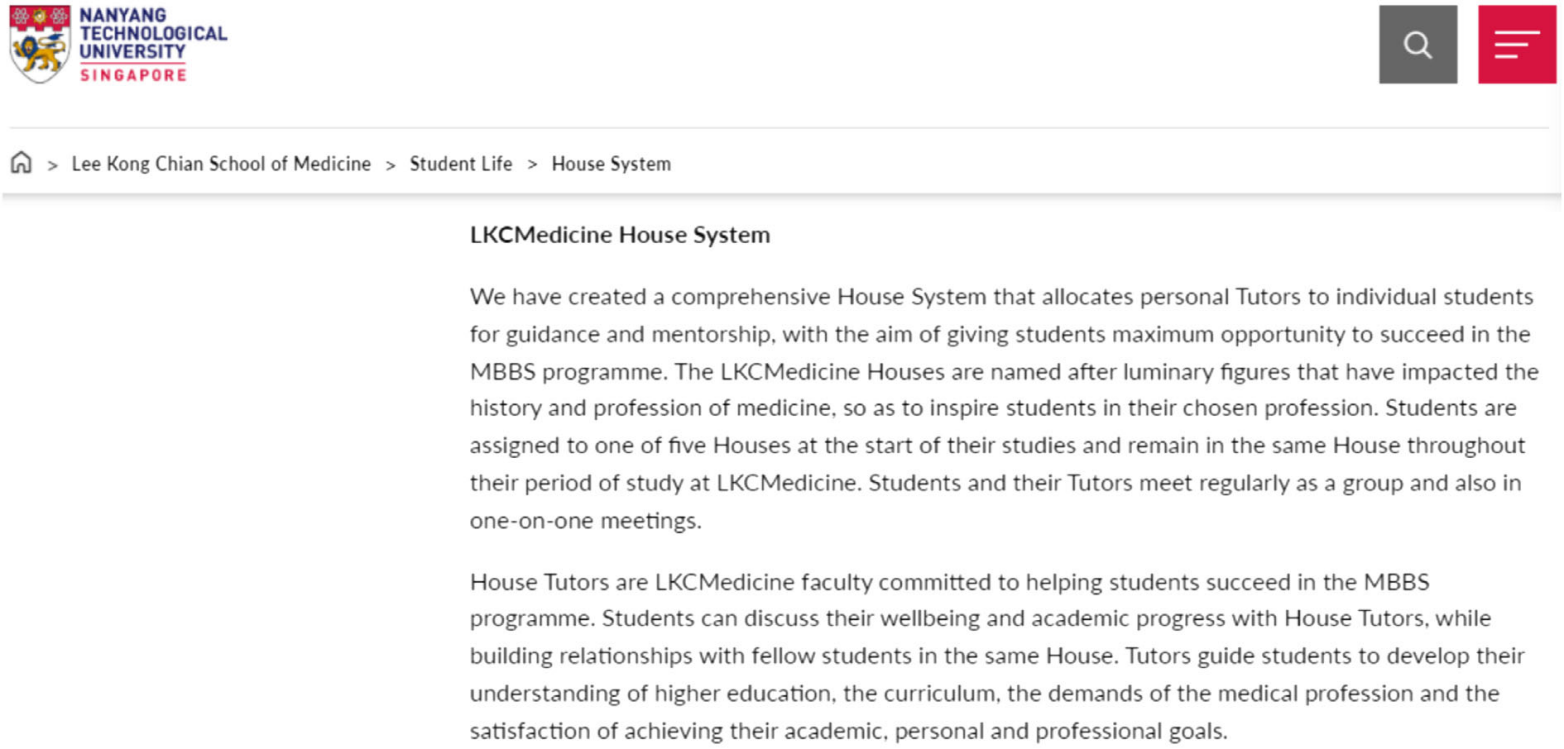
Screengrab of LKCMedicine ‘house system’ on its official website. Source: Lee Kong Chian School of Medicine, Nanyang Technological University. LKCMedicine House System. https://www.ntu.edu.sg/medicine/student‐life/house‐system (accessed 17 May 2023). [Color figure can be viewed at wileyonlinelibrary.com]

The institutional ‘texts’ clearly portrayed a sense of this promise, foregrounding a strong commitment towards support in their messaging. The medical school website prominently features its commitment to fostering a positive and supportive environment (Figure 1). Some illustrative examples were ‘… committed to helping students succeed’ and ‘students can discuss their well‐being and academic progress with house tutors’.

Students' expectations were reinforced by institutional processes, such as the clear outline of student support in the house system handbook for students, emphasising the commitment and support available through the house system and tutors (see quote below).

“**5**. The Lee Kong Chian School of Medicine will ensure that:

(ix) Academic and pastoral support is provided through the House Tutor system. Where necessary, additional support through the School or NTU will be offered, including study skills advice and counselling services”.

(Extract from LKCMedicine Student Agreement. *Note: The term ‘pastoral care’ and ‘support’ are used interchangeably in our day‐to‐day context*.)

In fact, students began their first weeks in the medical school by signing a ‘student agreement’ with their assigned house tutors, who used this process as a vehicle to explicitly ask what support the student may need and to explain that they were the first point of contacts for support. House meetings were also formally integrated into student timetables. Our sense from the interviews was that the students viewed the house tutors as part of the institutional support system, as their main point of contact for supporting their well‐being needs.

The expectations and promises of the role of the House System are also deeply entrenched within the organisation and school culture. The medical school's White Coat Ceremony is a case in point. This event has traditionally been organised as a grand affair, involving prominent guests and students' parents. During the committee meetings, the house system and house tutors take on a central role in the ceremony by helping students put on their white coats. The organisational structure extends to seating arrangements, where students are grouped according to their respective houses. One participant thought ‘it was a very natural decision … even if there was free seating, people would still probably sit by their House’ (P13). The house names are prominently displayed on the stage. In fact, the planning of the ceremony revolves heavily around the house system as seen in the recorded fieldnote below.

The members discussed intensely on whether to include the House Tutors' names on the screens (in the auditorium) when the students are presented with their white coat. The committee also deliberated on house logos, colours, and names to be shown on the two side screens of the auditorium, which reflected the prominence role of the House System and Identity in key school ceremonies. (Fieldnote, White Coat Ceremony committee meeting)

### 3.2 Tensions and mismatch between expectations and realities

While all these promises and early experiences were very positive, the expectations students formed were not always met. For example, some students felt like outsiders in a structure that was meant to be supportive, not always getting along with the other students in their house group, feeling ‘othered’ or ‘excluded’ (note that groups were pre‐organised by administrative and academic staff, and students stay in the same group for their time at medical school). As one participant put it, ‘it's hard to make friends this way, to make friends from people in that clique … their presence is very intimidating’ (P02).

In the house system handbook for students, the institutional discourse around the roles of house tutors framed them as advocates, someone who always acts on the behalf of the student, in the student's best interests. As one participant described his expectations on the house tutor's advocacy role, ‘I guess Prof. x would try his best to talk to Prof. y about this (to excuse the student from a mandatory class because of his personal circumstances) if I really wanted to push for it … I'm not sure how far he'll get. But I'm sure that Prof. x would definitely try his best’. (P03).

However, some students also experienced a mismatch between their expectation of house tutor as advocate and the actual support they received, feeling the student–tutor relationship was more transactional than relational. There was a sense that tutors were ‘just’ doing a job, rather than having a genuine interest in the student's personal development. Students with these perceptions felt a sense of misalignment between the institutional support framework, the perceived promises of engagement and commitment from tutors, and their experiences: ‘The house tutor system sometimes feels like they are just doing a job. I think is a bit unfortunate, right, uh, but, you know, they come in and say, okay, how are you doing at school? No problems, okay, sure, great. How are you doing at home? No problems, okay, great, I'll see you in three months. The camaraderie isn't really there’. (P07).

### 3.3 Sensemaking and reciprocity

Students engaged in a process of sensemaking to process their experiences with these mismatches. Students had to *interpret* and *make sense* of the promises and what the role of the house system and tutor actually meant for them. As one participant described a typical interaction with their house tutors, ‘it's nice to have someone advocate for you and will regularly check on you. (But), in reality, I think the relationship with a House Tutor differs from person to person. For myself, (my relationship with my tutor) is a bit superficial, in the sense that I will tell my House Tutor about how I am doing but it's not necessarily the avenue that I would choose to go through if I need help or anything’. (P11).

The misalignment in this psychological contract between the students' expectations of the tutor's role and their experiences then negatively impacted the tutor–student relationship. Students tried to make sense of the situation by adjusting their expectations of their perceived support from the school. For some, this meant a barrier to seeking help from the school and their house tutors. When they felt the psychological contract was too strained for repair, they reduced their engagement in well‐being activities and kept their relationship with their house tutors and official peer support groups merely transactional.

The reciprocity in the psychological contract between students and the house system was also continuously contested. Students wanted support from the school but did not want tutors who had demanding expectations of them. When they perceived tutors as overly demanding or rigid in their expectations, they were dissatisfied and their trust in the tutor and support system was eroded. One participant reflected on the role of the house tutor:

I think there's a few things they should do. So basically, if the students don't feel like you (are) worth their time or like, like they can't even open up or they can't even trust you then there's no point in you being their House Tutor, you get what I mean? (P03)

### 3.4 Agency, empowerment and legitimacy

Students demonstrated agency in their responses towards these tensions. For example, they set up a peer‐support initiative, ‘Housefam’, a play on the words house and family. Housefam is a near‐peer support structure that was initiated and implemented by students. Many felt more supported in their Housefam than the house group to which they had been assigned by the school.

Housefam is essentially a ‘workaround’, a strategy that students employed to navigate the perceived limits and constraints of the institutional well‐being system. As a ‘hidden’ support structure, Housefam remains invisible and officially unacknowledged in any institutional texts, policies and guidelines on the availability and provision of formal support within the medical school. Yet it is widely acknowledged as a support mechanism within the School, and the fact that the school funds some Housefam organised activities confers its legitimacy.

Something that started as a student‐led initiative, or workaround, is now a complementary part of the institutional practices, sometimes even held up as a model for how the official house system and tutor meetings should work. This is illustrated in one participant's comment, ‘if the House System can operate in a sense like how a Housefam do … I don't know whether like the school itself can do something like’. (P03).

There was a sense that the school sanctioned and supported the student‐led Housefam structure because of an (unvoiced) awareness that the ‘official’ house system did not meet every student's needs.

## 4 DISCUSSION

Previous studies have highlighted the complexity and dynamic nature of the role of expectations that medical students must contend with, from classroom settings to clinical training environments.^36^, ^37^, ^38^ Our study adds to this literature, providing insight on the role of expectations in shaping students' experiences of student support systems. Drawing on the psychological contract framework,^13^ our findings suggest that, where there was a mismatch between expectation and experience, students tended to disengage from the house system, engaging only minimally, to the extent they had to.^39^, ^40^

Our findings align with the general literature on the important role of expectation (mis)alignment, particularly in areas such as feedback, coaching and mentoring. For example, learners often expect timely, constructive feedback, but their readiness for feedback is affected by their expectation of the feedback.^41^ Similarly, misaligned expectations regarding the level of support from mentors or coaches can significantly impact learning and interpersonal relationships between mentors and their charges.^42^, ^43^, ^44^

None of the participants in this study completely left the tutor–student relationship (exit) probably because they were required to meet their house tutors regularly. However, they were not passive, just waiting for things to improve (loyalty) but instead engaged with an alternative support system developed by previous batches of students, the Housefam. The consequence of this was two co‐existing systems, the formal, school‐driven house system and the informal (but supported by the school) Housefam. Such workarounds are not uncommon and have been employed in various contexts such as to manage complexity or advocacy.^45^, ^46^ However, our study revealed an interesting finding. We did not observe any tensions between the house system and the Housefam. Instead, the two systems interacted synergistically, perhaps spurred by their shared goals of supporting student well‐being.

The co‐existence of two support systems suggests that a ‘one‐size‐fits‐all’ approach towards student support may not suffice, leading to a challenge for institutions. There is increasing attention on whether medical schools could and should do more in terms of student support. Institutions are likely to be closely scrutinised if they do not offer reasonable student support services to their students. Yet they are equally likely to be criticised for providing a system when students do not engage sufficiently. They are dammed if they do, and dammed if they do not (provide support).

However, some students do not want to engage with institutional support measures—possibly because they perceive seeking support as stigmatising or because of inherent limitations in the support system—or engage with them only until they build their own support systems. Other students may not have this capacity or sufficient networks to establish their own support system. Student support needs will also fluctuate over time. These points pose a dilemma for medical schools: How can an institution develop a system that fits the needs of all students across time? On balance, we argue that institutions should provide support measures and create a culture where help seeking is normalised and supported.

A feature of our context is that certain aspects of the student support system are mandatory (e.g. regular meetings with the house tutor). Mandatory well‐being activities are often viewed with disdain and scepticism by students who prefer autonomy in managing their own well‐being.^47^ However, a recent paper^48^ suggests that students actually welcome them. In fact, structured activities such as one‐on‐one interaction with a concerned representative of the institution can positively contribute to student well‐being.^49^ Existing literature on students' preferences of mandatory well‐being activities predominantly focuses on Western cultures and less so with regard to Asian students. Cultural differences may play a significant role in shaping these preferences. Further investigation into these cultural nuances can enrich our understanding of mandatory well‐being activities.

The support system under study in this paper was developed by the institution without student involvement. This ‘top‐down’ approach, which is the norm in all aspects of Singapore education and society, may have at least partially contributed to the development of the alternative, student‐led ‘Housefam’ system. The broader message is that support systems may be most effective if students, the users, are given agency in respect of being involved in the design, or re‐design, of student support structures.^50^ Empowering students^51^ to be able to take charge of their own well‐being may be considered a type of co‐creation, the active and collaborative involvement of the learners in the design and development of educational practices,^52^ in this case student support. Enabling student empowerment means medical schools must feel comfortable to admit that they may not know everything about how best to support students and be open to bottom‐up innovations from students to supplement (or even replace) top‐down approaches. Student expectations of support may be different when these are not shaped solely by perceptions of institutional promises (expectations) but also by acknowledging that they are partners in planning and enacting support systems. Actively engaging with students to design student support may also enrich student experiences.

## 5 IMPLICATIONS

It is important to examine the way a school presents itself to the public through the school website and communication materials.^53^ Our data suggest that these do not just serve as marketing to attract the potential applicants but also play an important role in shaping students' expectations; in this case of the support, they will receive once at medical school. In line with psychological contract theory, any mismatch between expectations and experiences may negatively impact students' engagement with institutional support mechanisms. Perceived breaches of psychological contracts can lead to feelings of betrayal, erosion of trust in the institution, and ultimately reducing their motivation to engage with institutional support mechanisms. Therefore, a medical school's promotional materials and communications should not be viewed as mere marketing resources. They also play an important role within the educational context in setting out the psychological contracts between the school and their students.

Our study also has research implications. We do not know about student expectations of their own role in the house system. Did they primarily view it as uni‐directional, with the house tutor being primarily responsible or did they also see themselves as having agency within the relationship? This requires further exploration. Also, our study represents a snapshot in time. The data suggest that the relationship between students and the support system, the house system in this case, evolves over time and will be influenced by other factors (e.g. what the student needs and what other support is on offer). Longitudinal follow‐up studies examining the relationship between students and support systems from entry to medical school to graduation are required to extend knowledge on this topic.

### 5.1 Strengths and limitations of this study

The notion of psychological contract is widely used in education more generally, and its use allowed us to access a wider literature, which was useful in formulating our thoughts about the messages from this study.^54^, ^55^ It allowed us to present and analyse our data to maximise transferability to other contexts. It is a theory that may be useful in any area of medical education where students' expectations may play a significant role in influencing relationships and outcomes. However, conceptual lenses are operators that favour and allow certain investigations and conclusions and, at the same time, block others. In other words, another lens may have highlighted different aspects of the data.

Our open invitation drew in participants from three of the five houses in our medical school. It is possible that the challenges we identified pertain to specific houses/house tutors rather than are common challenges across the whole house system. Given the diversity of individual participants, we are inclined towards the latter.

We recognise that students' expectations are context and culture‐dependent.^56^, ^57^ These factors may not be fully captured in our study, and it may be that students in other contexts and cultures have different expectations. Yet, on the other hand, although this paper is embedded in the Singapore context, its focus and messages are relevant to medical schools in other contexts that have adopted a personal tutor system for well‐being/student support. While the rich description we provided in this study will also help in transferability, providing information to resonate with colleagues working in their own contexts and settings, we call for future cross‐cultural research to elucidate how student (and tutor) expectations influence experiences of support. Finally, our focus was student views: We call for other studies examining the views of other key stakeholders in student support, including the perspectives of those providing support.

## 6 CONCLUSION

Medical students' expectations play an important role in shaping their experiences of school‐wide well‐being programmes. A deeper understanding of the role of expectations can inform the design and development of support structures that meet student needs.

## AUTHOR CONTRIBUTIONS

**Emmanuel Tan:** Conceptualization; investigation; writing—original draft; methodology; writing—review and editing; project administration. **Erik Driessen:** Conceptualization; supervision; writing—review and editing; methodology; investigation. **Janneke Frambach:** Conceptualization; investigation; writing—review and editing; supervision; methodology. **Jennifer Cleland:** Conceptualization; investigation; writing—review and editing; methodology; supervision. **Grainne Kearney P:** Conceptualization; investigation; writing—review and editing; methodology; supervision.

## CONFLICT OF INTEREST STATEMENT

The authors declare that they have no competing interests.

## ETHICS STATEMENT

The study protocol (IRB‐2022‐259) was approved by the Institutional Review Board of Nanyang Technological University. Informed consent was obtained from all participants.

## Supporting information


**Appendix S1.** Interview guide.

**Appendix S2.** Texts.

## Data Availability

The data that support the findings of this study are available from the corresponding author upon reasonable request.
