# Supplementary material for: How do medical students' expectations shape their experiences of well‐being programmes?
Source: Med Educ. 2024 Sep 24;59(3):309–17. doi: 10.1111/medu.15543 (PMC11789832; doi:10.1111/medu.15543)
Supplement: Supplementary file 1 — Appendix S1. Interview guide. [file MEDU-59-309-s002.docx]

**Appendix 1: Interview guide**

1. What is your role in the House System?
2. How has your experience of the House System been so far?
3. How clearly do the House System’s policies and local documents spell out what is required for students and staff? And what do you think is the role of the House Tutor in relation to the following policies?
4. What are your views regarding the practicalities of the House System?
5. What do you think is the role of a House Tutor?
6. How strongly do you identify as a member of your House?
7. What is your take on the ‘House family’*?
8. Many clinical students tend to prioritise their academic over wellbeing activities. What are your views on this?
9. What are the main strengths and weaknesses of the House System?

* House Family is an informal peer support structure created by students alongside the main, institutional system.
