# Supplementary material for: How do medical students' expectations shape their experiences of well‐being programmes?
Source: Med Educ. 2024 Sep 24;59(3):309–17. doi: 10.1111/medu.15543 (PMC11789832; doi:10.1111/medu.15543)
Supplement: Supplementary file 2 — Appendix S2. Texts. [file MEDU-59-309-s001.docx]

**Appendix 2** **- Texts**

1. House System Guide for Students
2. Student Agreement
3. Concern Form for Medical Students
4. Mitigating Circumstances Form
5. Leave of Absence Application Form
6. Interruption of Studies Application Form
7. Minutes of White Coat Ceremony
8. Minutes of Student Wellbeing Committee meetings
9. Minutes of Student-Staff Liaison Committee meetings
